# Analysis of dendritic cells in human lymphoid organs

**DOI:** 10.1186/1479-5876-10-S3-O3

**Published:** 2012-11-28

**Authors:** Gordon F Heidkamp, Nathalie Eissing, Lukas Heger, Robert Cesnjevar, Arndt Hartmann, Johannes Zenk, Evelyn Ulrich, Andreas Mackensen, Gerold Schuler, Burkhard Schauf, Falk Nimmerjahn, Diana Dudziak

**Affiliations:** 1 Dept. of Dermatology, Laboratory of Dendritic Cell Biology, University Hospital Erlangen, Germany; 2 Dept. of Pediatric Heart Surgery, University Hospital Erlangen, Germany; 3 Dept. of Pathology, University Hospital Erlangen, Germany; 4 Dept. of Otorhinolaryngology, University Hospital Erlangen, Germany; 5 Dept. of Hemato-Oncology, University Hospital Erlangen, Germany; 6 Dept. of Dermatology, University Hospital Erlangen, Germany; 7 Dept. of Obstetrics and Gynecology, Sozialstiftung Bamberg, Bamberg, Germany; 8 Friedrich-Alexander Universität Erlangen-Nürnberg, Chair of Genetics, Dept. of Biology, Erlangen, Germany

## Introduction

Dendritic Cells (DCs) are important regulators of immune responses. In our previous studies we found differential antigen presentation capacities of murine DC subpopulations using an in vivo antigen targeting system [1]. In contrast to murine DCs, the functional role of human tissue DCs is largely unknown.

## Aim

We are focussing on the characterization of DC subpopulations directly isolated from human lymphoid tissues to understand their functional role in the human immune response.

## Patients and methods

Human tissues (thymus, spleen, bone marrow, tonsils, cord blood, peripheral blood, together around 300 samples) were received from otherwise healthy individuals. For our studies we performed 6 color confocal immunofluorescence analyses, and 10 color FACS and cell sort analyses for the study of 284 cell surface molecules (Lyoplate assay). We further investigated the DC’s antigen uptake properties and analyzed the RNA expression by microarrays.

## Results

The percentage of the three main DC subpopulations of mDC1, mDC2 and pDCs was varying depending on the tissue analyzed, indicating different functional roles of the DC subpopulations. Only very view cell surface molecules were uniquely expressed on the different DC subpopulations. Further, future potential antigen targeting receptors of the C-type lectin and Fc receptor family were investigated. Depending on the targeting antibody CD4 or CD8 T cell responses could be initiated. Our microarray data together suggest differential antigen presentation capacities of pDCs, mDC1, and mDC2 cells.

## Conclusion

With cutting edge technologies we have characterized directly isolated human DC subpopulations.

## Competing Interests

D.D. was a fellow of the ‘Förderkolleg’ of the Bavarian Academy of Sciences.

## Acknowledgements

This study (D.D.) was partly supported by DC-Thera, the German Research Foundation (SFB643-TPA7 and DU548/2-1, GRK1660) and BayGene.
